# Correction: Nedelcu et al. Low-Resolution Precoding for Multi-Antenna Downlink Channels and OFDM. *Entropy* 2022, *24*, 504

**DOI:** 10.3390/e25030445

**Published:** 2023-03-03

**Authors:** Andrei Stefan Nedelcu, Fabian Steiner, Gerhard Kramer

**Affiliations:** 1 Optical and Quantum Laboratory, Huawei Munich Research Center, 80992 Munich, Germany; 2 Institute for Communications Engineering, Technical University of Munich (TUM), 80333 Munich, Germany

## 1. Error in Figure

In the original publication [1], there was a mistake in Figure 1 as published. The labels of the output signals x_1_[t]…x_N_[t] should appear at the output of the power amplifier as transmit waveforms. The corrected version of Figure 1 appears below. 

## 2. Text Correction

There was an error in the original publication. “Blind detector” is incorrect and should be replaced throughout with “data aided detector”.

1. A correction has been made to **Abstract:**

“The information rates are computed for pilot-aided channel estimation and data-aided channel estimation.”

2. A correction has been made to **1. Introduction, *1.2. Discrete Signaling and OFDM*, Paragraph Number 1**:

“For this purpose, we consider two types of channel estimation at the receivers: pilot-aided channel estimation via pilot-aided transmission (PAT) and data-aided channel estimation.“

3. A correction has been made to **1. Introduction, *1.3. Contributions and Organization*, Bullet Point Number 4**:

“We develop an auxiliary channel model to compute achievable rates for pilot-aided and data-aided channel estimation. The models let one compare modulations, precoders, channels, and receivers;”

4. A correction has been made to **4. Performance Metrics, *4.1. Achievable Rates*, Paragraph Number 2**:

“We study the GMI of two non-coherent systems: classic PAT and data-aided channel estimation. For both systems, we apply memoryless signaling with the product distribution”

5. A correction has been made to **4. Performance Metrics, *4.1. Achievable Rates*, Paragraph Number 5**:

“For the data-aided detector we replace 𝑆_𝑝_ with 𝑆 in (20).”

6. A correction has been made to **4. Performance Metrics, *4.1. Achievable Rates*, Paragraph Number 6, Bullet Point 3**:

“For the data-aided detector, in (21) we replace S_𝑝_ with the set of all index pairs (ℓ,𝑚), and we replace 𝑆_𝑝_ with 𝑆;”

7. A correction has been made to **4. Performance Metrics, *4.1. Achievable Rates*, Paragraph Number 5, Bullet Point 4:**

“For the data-aided detector we set S_𝑝_ = ∅ in (22);”

8. A correction has been made to **4. Performance Metrics, *4.2. Discussion*, Paragraph Number 1**:

“Third, as 𝑆 grows, the channel estimate of the data-aided detector becomes more accurate and the performance approaches that of a coherent receiver. Related theory for PAT and large 𝑆 is developed in [49]. However, the PAT rate is generally smaller than for a data-aided detector because the PAT channel estimate is less accurate and because PAT does not use all symbols for data.”

9. A correction has been made to **4. Performance Metrics, *4.2. Discussion*, Paragraph Number 2**:

“We remark that blind channel estimation can approach the performance of data-aided receivers for large 𝑆. Blind channel estimation algorithms can, e.g., be based on high-order statistics and iterative channel estimation and decoding.”

10. A correction has been made to **5. Numerical Results, Paragraph Number 2:**

“The average GMIs for Systems A–C were computed using 𝑆 = 256, 𝐵 = 200, and a data-aided detector. The coded results of System D instead have 𝑆 = 1584 symbols to fit the block structure determined by the LDPC encoder. For System D we considered both PAT and a data-aided detector.”

11. A correction has been made to **5. Numerical Results, Paragraph Number 7**:

“The solid curves are for data-aided channel estimation and the dotted curves show the performance of PAT when the fraction of pilots is 𝑆_𝑝_/𝑆 = 10%.”

12. A correction has been made to **6. Conclusions, Paragraph Number 1**:

“The performance was analyzed by computing the GMI for two auxiliary channel models: one model for pilot-aided channel estimation and a second model for a data-aided channel estimation.”

The authors state that the scientific conclusions are unaffected. This correction was approved by the Academic Editor. The original publication has also been updated.

## Figures and Tables

**Figure 1:**
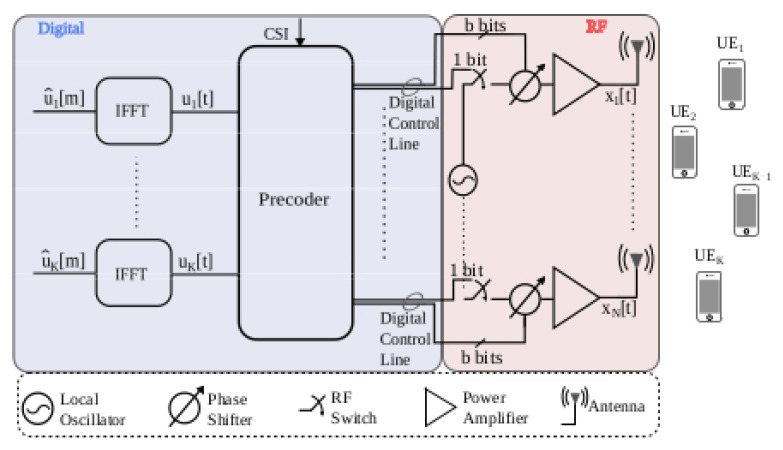
Multi-user MIMO downlink with a low resolution digitally controlled analog architecture.
